# Effect of household and village characteristics on financial catastrophe and impoverishment due to health care spending in Western and Central Rural China: A multilevel analysis

**DOI:** 10.1186/1478-4505-9-16

**Published:** 2011-04-06

**Authors:** Wuxiang Shi, Virasakdi Chongsuvivatwong, Alan Geater, Junhua Zhang, Hong Zhang, Daniele Brombal

**Affiliations:** 1 Unit of Epidemiology & Health Statistics, Guilin Medical University, Guilin 541004, China; 2 Epidemiology Unit, Faculty of Medicine, Prince of Songkla University, Hat Yai, Songkhla 90110, Thailand; 3 Health Human Resources Development Center, Ministry of Health, Beijing 100097, China; 4 Office of Cooperation Development of Italian Embassy in China, Beijing 100097, China

## Abstract

**Objective:**

The study aimed to examine the effect of household and community characteristics on financial catastrophe and impoverishment due to health payment in Western and Central Rural China.

**Methods:**

A household survey was conducted in 2008 in Hebei and Shaanxi provinces and the Inner Mongolia Autonomous Region using a multi-stage sampling technique. Independent variables included village characteristics, household income, chronic illness status, health care use and health spending. A composite contextual variable, named village deprivation, was derived from socio-economic status and availability of health care facilities in each village using factor analysis. Dependent variables were whether household health payment was more than 40% of household's capacity to pay (catastrophic health payment) and whether household per capita income was put under Chinese national poverty line (1067 Yuan income per year) after health spending (impoverishment). Mixed effects logistic regression was used to assess the effect of the independent variables on the two outcomes.

**Results:**

Households with low per capita income, having elderly, hospitalized or chronically ill members, and whose head was unemployed were more likely to incur financial catastrophe and impoverishment due to health expenditure. Both catastrophic and impoverishing health payments increased with increased village deprivation. However, the presence of a village health clinic had no effect on the two outcomes, nor did household enrollment in the New Rural Cooperative Medical Scheme (national health insurance).

**Conclusions:**

Village deprivation independently increases the risk for financial hardship due to health payment after adjusting for known household-level factors. This suggests that policy makers need to view the individual, household and village as separate units for policy targeting.

## Background

Financial problems ensue when individuals or households have to use a large fraction of their resources to pay for health care, which can threaten the living standards either in the short term, as the immediate consumption of other goods and services must be sacrificed, or in the long term, as assets must be diverted, savings depleted or debt accumulate[1]. There are two main approaches to measuring serious financial burden. One indicator is financial catastrophe, when household spending on health care (as a proportion of the total household income) surpasses a pre-specified fraction, Z. The idea is that a household should have at least 1-Z of their income to spend on things other than health care. Medical spending above Z would disrupt and cause financial catastrophe[2]. Another indicator is impoverishment. This method, which sets the minimum in terms of an absolute level of income, entails that health expenses should not push a household into poverty, and not further deepen financial difficulty to those under the poverty line[2].

Financial catastrophe and impoverishment due to health spending are real and sizable worldwide problems, especially in the developing countries where out-of-pocket (OOP) payment is the social norm. WHO recently published estimates suggesting that, worldwide, more than 44 million households face financial catastrophe, and that about 25 million households are pushed into poverty because of medical expenditure annually[3]. However, OOP payment is not the only important factor related to these financial difficulties. The triad of poverty, health-service access and use, and the failure of social mechanisms to pool financial risks account for most of the variation across countries[4]. Generally, poorer households tend to spend a higher percentage of their budgets on health care than do wealthier households, but this pattern does not hold universally across countries[5]. Other household characteristics, such as households headed by an elderly, disabled or unemployed person, and families with reduced access to health insurance and a greater proportion of elderly members, as well as those with chronically ill or hospitalized members, are at a high risk of catastrophic and impoverishing expenditure[6,7].

In the past, because these two adverse financial outcomes have been measured at the household level, their influencing factors have also been examined only at this level. To our knowledge, most relevant studies ignored the impact of contextual variables on these financial burdens. A contextual variable holds that all members living in the same community share the same values, which may be non-modifiable, for example, distance from village to county seat, or modifiable, such as availability of health care services[8,9]. There may also be cross-level correlation[10]; for example, poor people often live in deprived villages. Ignoring all contextual variables, modifiable factors that can have an effect on the whole community may be missed. If an outcome variable, such as individual health status or medical financial burden, were correlated within a neighbourhood, the single-level analysis using common regression methods would underestimate the standard errors for contextual effects and give biased results[11]. Currently, multilevel analysis based on linear mixed models can be used to delineate these complex relationships and give correct estimation of the effect of factors at different levels.

China is one of the countries with a high rate of OOP health payment (60%)[12].

To reduce the financial burden on rural residents, the Chinese government initiated the New Rural Cooperative Medical Scheme (NRCMS) in 2003. It is a government-run, voluntary, community-based, and cost-sharing medical insurance program, covering a county (0.5-1 million people) as a unit. This scheme requires the participating household to pay a part of the premium, which includes a household contribution of 20 Yuan (RMB), a central government subsidy of 50 Yuan and 50 Yuan from the local government per capita per year in 2008. Local governments are free to choose the benefit package and administrative arrangement of their own NRCMS according to local needs, thus, benefit packages vary from one county to another. To supplement the NRCMS, the Medical Financial Assistance (MFA) was also established at the same year, which is supposed to provide assistance mainly for the officially designated poor.

Apart from the reports provided by the offices responsible for NRCMS and other insurance schemes in China, there have been only a few independent and rigorous assessments of the program. Moreover, there is no consensus on whether the current health prepayment schemes (NRCMS and MFA) in rural China prevent effectively their members from catastrophic and impoverishing health payment.

Our study focuses on the Western and Central Rural regions of China, which comprise a rural population of approximately 500 million[13], where poverty is more prevalent than in other parts of China[14]. A recent household survey on the health financing system in rural China was conducted[15]. In the current communication, we revisit the data set examining the contextual effects of village variables to serve the above needs.

The specific objectives of this study are to identify the factors at village and household levels that are independently associated with financial catastrophe and impoverishment due to health payment in the study areas.

## Methods

### Study design

A cross-sectional community household survey was conducted from May to August 2008 in Hebei and Shaanxi provinces, and the Inner Mongolia Autonomous Region, which represent Western and Central China. From each selected province, two counties with population, area and per capita incomes close to the average provincial values were chosen. Additionally, counties which did not have the New Rural Cooperative Medical Scheme (NRCMS, a government-run voluntary community-based insurance[15] available to its residents, or where the scheme had been introduced for less than one year, were excluded from selection. In each selected county, the towns were classified as better-off or worse-off, depending on their average annual income and from each group, one town was randomly selected. In each selected town, all natural villages were grouped into two classes: close, and far, depending on their distance from the town centre. In each class, three natural villages were randomly selected and finally, 45 households from each natural village were chosen by simple random sampling.

### Study variables

The variables and their definitions where required, are listed as follows:

### Dependent variables

All dependent variables were at the household level.

#### (1) Household with catastrophic health payment

A household in which its OOP payments in the year prior to the interview caused the household to cross a pre-specified share of household living standards[2]. The WHO threshold, 40% of household capacity to pay,[16] was utilized to measure catastrophic health payment in this study.

#### (2) Household impoverished by medical spending

*A *household that originally had its per capita income above the poverty line, but consequently fell below the line after health payment[2]. The Chinese national threshold income of 1,067 Yuan per year per capita in 2008 was adopted[17].

### Independent variables

#### Household level independent variables

##### Household head

(1) *Age*, (2) *sex*, (3) *ethnicity*, (4) *educational attainment*, (5) *religion*, (6) *marital status*, (7) *occupation, the unemployed of which was defined as the one who can't do any job *because of age, disability, sickness or things like that.

##### Health status, living standards, utilization of medical care and health payment

(8) *Number of patients with serious chronic illness: *The definition of serious chronic condition was modified based on The Family and Medical Leave Act (FMLA)[18], which we defined as a health problem which lasts, or is expected to last, for at least 12 months, resulting in incapacity and treatment for more than 10 days accumulated within the year prior to the interview, and including disability.

(9) *Household members hospitalized within 12 months prior to the interview*.

(10) *Household NRCMS participation status*.

(11) *Household living standard*: For countries like China, where there is a culture of a high rate of saving money (53% of total income)[19], non-essential expenditure is likely to be distorted by this propensity to save, hence household consumption expenditure is considered as not a good proxy of household economic status[20,21]. Therefore, household income was used to measure living standard in this study. The annual household income was defined as the aggregate of household income from production (10 categories), wage incomes of household members, transfer income (gifts, pensions, remittances, welfare) and property income (interest, rent)[20]. Food expenditure data were also collected with a recall period of three months. This variable was subtracted from total income to get the effective income, which was used to calculate financial catastrophe due to health spending.

(12) *Health payment*: This was defined as the net out-of-pocket health payment, as a share of household resources, and included payments for outpatient care, self-prescribed medication and traditional medicine with a recall period of one month, and inpatient care payment with a recall period of one year preceding the survey[22]. Considering the different recall time periods between outpatient and inpatient services to calculate catastrophic health payment and impoverishment, data on outpatient cost per year was obtained by payments for outpatient cares recalled multiplied by 12 months. Insurance reimbursement was deduced from the reported household payments, as were indirect expenses related to non-medical items, such as transportation, special foods, and accommodation.

#### Village level independent variables

(13) *Location of village: *plains or mountains

(14) *Village average income*: defined as the per capita annual net income in a village during the year prior to the interview.

(15) *Village adult illiteracy rate: *defined as the percentage of population, aged 15 and above, unable to read and write a short simple statement on one's everyday life.

(16) *Availability of village health clinic*

(17) *Distance from village to town health center*

(18) *Distance from village to county hospital*

### Data Collection

The questionnaire for the household survey was modified from that used in the Fourth National Health Service Survey in 2008 in China to suit the needs of the study[23]. It consisted of four sections: a) household composition, status of household participation in NRCMS, household income and expenditure; b) age, sex, ethnicity, religion, marital status, highest education level attained, employment status and self-reported chronic illness status of household members; c) health utilization and associated costs that occurred within 30 days prior to the interview; d) hospitalization during the year prior to the interview. To validate the questionnaire, input from public and scientific advisory committees was obtained, and 2 pilot studies were conducted before the formal survey.

Thirty-six medical students from the College of Medicine at Xianjiaotong University and other medical schools from the provinces under investigation were trained to be data collectors under the supervision of three senior experts. Information on village average income, village adult illiteracy rate, availability of health clinics, and distances from village to town health center and county hospital were obtained from village officials. The household survey data were collected by face-to-face interviews with the household head. Respondents were given full explanation of the research purpose before being invited to participate and, after they gave their informed consent, the interview was conducted. The data were collected from May to August 2008.

### Data management and statistical analysis

In addition to variables directly collected from the interview, other indicators were obtained by data manipulation. The proportion of household members with serious chronic illness in a household was arbitrarily divided into three levels, i.e. 0, >0 to ≤0.5, and >0.5. The same procedure was followed with the proportion of the elderly (over 65 years) in a household, whereas the household per capita income was grouped into quartiles.

Since the majority of village-level variables were highly correlated, putting more than one of them in the model would cancel the effect of the others, which may be misleading. In order to retain the influence of all these variables in the model, factor analysis was conducted. A factor score was computed, as a single variable, representing the relative deprivation level of the community. The dichotomous variables such as availability of health post and location of village were not included in the composition of index by factor analysis.

Apart from descriptive statistics and simple cross tabulation, a multilevel, mixed-effects logistic regression model was applied to identify the determinants of a household incurring catastrophic payment and impoverishment. These dependent variables were set at the first level (household) nested within the second level (village). Since each village represented villages in the population, the household in each selected village was modeled to share a common baseline odds of having financial difficulty, and this is a random variable so constitutes the random effect part of the model. The explanatory variables, such as the aforementioned household and village characteristics, have fixed influences on the odds of having financial difficulty, and this constitutes the fixed effects part of the model. To check the level of correlation of the outcome variable, the intra-class correlation (ICC) was also computed. All data were analyzed with R software version 2.9[24].

### Ethical approval

The proposal of this study was approved by the Ethics Committee of the Faculty of Medicine, Prince of Songkla University, Thailand, and further endorsed by the Health and Human Resource Development Center of the Ministry of Health, P.R. of China, before the research was carried out.

## Results

A total of 3,334 residents from 3,340 households and 97 natural villages in six counties were interviewed, with a response rate of 99.8%. Data on 15 (0.45%) households were discarded due to error and incomplete information. The distribution of the surveyed households by county is shown in Table 1.

**Table 1:** Distribution of households by county

Province	County	Households N (%)
Hebei	Zaoqiang	649 (19.6)
	Jinxian	601 (18.1)

Inner Mongolia Autonomous Region	Aohanqi	491 (14.8)
	Ningcheng	580 (17.5)

Shaanxi	Jingbian	498 (15.0)
	Dingbian	500 (15.1)

**Total**		3,319 (100)

Table 2 presents the household, village and county characteristics. Most household heads were middle-aged, male, farmers, married, of the Han ethnicity and claiming no religious affiliation. Over 90% of the households participated in NRCMS. More than half of the villages had a health post. The median distance from the village to the town health center and county hospital was approximately five and 50 kilometers, respectively.

**Table 2:** Household and village characteristics

Variables		Values
**Independent Variables**		

**Household level - household head characteristics (N = 3,319)**		
Gender	Male (%)	83.9
	Female (%)	16.1
Age (Mean, SD^†^)		49.9 (±12.2)
Ethnicity	Han (%)	96.1
	Other (%)	3.9
Education level	None (%)	19.0
	Primary school (%)	39.3
	Junior high school (%)	35.1
	Senior high school or above (%)	6.7
Occupation	Farmer (%)	85.6
	Non-farmer (%)	8.4
	Unemployed (%)	5.8
**Household level - income, health status, insurance status and hospitalization (N = 3,319)**		
Per capita income (1,000 Yuan, median and IQR^‡^)		4.9 (3.3-7.4)
Size (Mean, SD)		3.3 (±1.4)
Proportion of members aged over 65 (%)		0.1 (±0.3)
Proportion of Members aged under 5 (%)		0.03 (±0.09)
Members with serious chronic illness (%)		31.0
Hospitalized members (%)		17.1
NRCMS participant (%)		91.2
**Village level (N = 97)**		
Number of households, median and IQR		23.8 (14.1-36.4)
Per capita annual income (×1,000 Yuan, Mean, SD)		2.0 (±1.3)
Adult illiteracy rate (%)		25.8 (±14.3)
Distance from village to town health center (Kilometers, median and IQR)		6 (3.0-10.0)
Distance from village to county hospital (Kilometers, median and IQR)		48 (29-75)
With village health clinic (%)		53.6
Mountainous village (%)		45.4
**Dependent Variables**		
Average percentage of catastrophic health payment(N = 3,319)		18.4
Average percentage of households impoverished by health spending (N = 3,077)*		12.0

Δ NRCMS: New Rural Cooperative Medical Scheme

* Number of non-poor households before health payment

† SD: Standard deviation

‡ IQR: Inter-quarter Range

### Correlation among village variables and the result of factor analysis

Out of the six village variables, four continuous variables, namely average village income, village adult illiteracy rate, distance from village to town health center, and distance from village to county hospital, were highly correlated. The correlation matrix among these four variables is displayed in Table 3. The screeplot of these four variables suggested only one underlying factor, village deprivation, of which its score was further divided into quartiles from most deprived (1) to least deprived (4). The correlation coefficient of location of village with village deprivation is 0.84, which may cause multicollinearity problems in analysis, so location of village was excluded in the models of the determinants of catastrophic and impoverishing health payments.

**Table 3:** Correlation matrix among village variables and factor score (village deprivation)

	Factor (village deprivation)	Village average income (Yuan)	Village adult illiteracy rate (%)	Distance from village to town health center (Km)	Distance from village to county hospital (Km)
**Factor** **(village deprivation)**	1				
**Village average income**	0.94	1			
**Village adult illiteracy rate**	-0.77	-0.62	1		
**Distance from village to town health center**	-0.7	-0.55	0.54	1	
**Distance from village to county hospital**	-0.76	-0.65	0.45	0.45	1

All p-values were ≤ 0.05

### Incidence of catastrophic and impoverishing health payment by all subgroups

Cross tabulation of the incidence of catastrophic and impoverishing health payment by all independent variables gave a similar conclusion. All variables except NRCMS and availability of health clinic in the village were significantly associated with both outcome variables. Household size was associated with catastrophic health payment, but not with medical impoverishment (Table 4).

**Table 4:** Incidence of catastrophic and impoverishing health payment by all subgroups

Variables	Households with catastrophic health payment (N = 3,319) n (%)	P-value*	Households impoverished by health payment (N = 3,077)^Δ^ N (%)	P-value
**Village-level variables**				
Village deprivation (Quartile)		<0.001		<0.001
Most deprived	109 (30.7)		68 (22.2)	
2nd	217(28.3)		142 (20.5)	
3rd	159 (15.3)		99 (9.9)	
Least deprived	130 (11.2)		59 (5.5)	
Availability of a health post		0.866		0.109
No	223 (18.7)		146 (13.3)	
Yes	392 (18.4)		222 (11.2)	
Location of village				
Mountainous	237 (28.0)	<0.001	157 (20.7)	<0.001
Plains	378 (15.3)		211 (9.1)	
**Household-level variables**				
Household per capita income (Quartile)		<0.001		<0.001
Lowest	355 (42.9)		222 (36.2)	
2^nd^	165 (19.9)		106 (13.2)	
3^rd^	67 (8.1)		31 (3.8)	
Highest	26 (3.1)		8 (1.0)	
NRCMS participation status		0.409		0.569
No	42 (16.4)		25 (10.6)	
Yes	573 (18.7)		343 (12.1)	
Proportion of members with serious chronic illness		<0.001		<0.001
0	194 (8.5)		121 (5.6)	
≤0.5	289 (36.2)		177 (24.8)	
>0.5	132 (57.9)		70 (37.8)	
Hospitalized members		<0.001		<0.001
No	365 (13.3)		216 (8.4)	
Yes	250 (44.2)		152 (29.7)	
Household size		0.004		0.123
≤3	393 (20.0)		210 (11.7)	
4-6	212 (16.0)		151 (12.0)	
>6	10 (29.4)		7 (24.1)	
Proportion of members aged over 65		<0.001		<0.001
0	350 (13.7)		221 (9.1)	
≤0.5	148 (28.1)		91 (19.3)	
>0.5	117 (49.6)		55 (33.7)	
Occupation of household head		<0.001		<0.001
Farmer	483 (17.0)		303 (11.4)	
Non-farmer	22 (7.9)		10 (3.7)	
Unemployed	110 (57.3)		55 (41.0)	

Δ Number of non-poor households before health payment; * Chi-square test of association

### Estimation of influence of household and village variables on financial catastrophe and impoverishment due to health payment

Tables 5 and 6 show odds ratios of various independent variables from the univariate analysis, multi-variate logistic regression, and finally mixed effect logistic regression. The p-values in both tables indicate the influences of these variables on the three models are similar. The results from the latter two types of regression were the same as that from univariate analysis due to the low level of confounding by the independent variables. The mixed effects logistic regression model gave estimates which were close to those from the multi-variate logistic regression model due to low level of intraclass cluster (in this context - intraclass correlation (ICC = 0.05 and 0.06, respectively)) and relative larger number clusters (97 villages). Both financial catastrophe and impoverishment decreased as the village deprivation quartile increased, with a significant dose-response relationship (p < 0.01), and households in the highest (least deprived) quartile were less likely to incur these financial hardship than those in other quartiles. The presence of village health posts had no relationship with these two indicators and neither did household enrollment in NRCMS (data not shown). At the household level, the household per capita income had a very strong independent inverse association with both financial catastrophe and impoverishment due to health payment. Households with larger proportions of elderly members were more likely than others to incur catastrophic and impoverishing health expenditure, as were households with a larger proportion of members having serious chronic illness and households with hospitalized members. Larger households were more likely to escape financial catastrophe than smaller households. This phenomenon was not observed in the analysis of impoverishment due to medical payment. Households with an unemployed household head were more likely to incur financial catastrophe rather than impoverishment.

**Table 5:** Determinants of catastrophic health payment

Variables	Univariate analysis		Logistic regression		Mixed effected logistic regression	
	
	Crude OR (95% CI)	P-value^Δ^	Adjusted OR (95% CI)	P-value^Δ^	Adjusted OR (95% CI)	P-value**
**Village-level variables**						
Village deprivation (Quartile)		<0.001*		<0.001*		<0.001*
2nd vs. Lowest deprived	0.89 (0.68-1.17)		1.24 (0.83-1.85)		1.19 (0.73-1.96)	
3rd vs. Lowest deprived	0.41 (0.31-0.54)		0.67 (0.45-0.99)		0.64 (0.40-1.04)	
Highest vs. Lowest deprived	0.28 (0.21-0.38)		0.41 (0.27-0.62)		0.39 (0.24-0.64)	
Availability of health clinic		0.866		0.34		0.291
Yes vs. No	0.98 (0.81-1.17)		1.14 (0.87-1.5)		1.21 (0.85-1.70)	
**Household-level variables**						
Household per capita income (Quartile)		<0.001*		<0.001*		<0.001*
2nd vs. Lowest	0.33 (0.27-0.41)		0.34 (0.26-0.45)		0.33 (0.25-0.44)	
3rd vs. Lowest	0.12 (0.09-0.16)		0.12 (0.09-0.17)		0.12 (0.08-0.16)	
Highest vs. Lowest	0.04 (0.03-0.07)		0.04 (0.02-0.06)		0.04 (0.02-0.06)	
Serious chronic illness proportion		<0.001*		<0.001*		<0.001*
≤0.5 vs.0	6.15 (5.01-7.57)		4.11 (3.2-5.29)		4.19 (3.24-5.42)	
>0.5 vs.0	14.93 (11.04-20.18)		8.71 (5.99-12.68)		8.99(6.11-13.22)	
Member hospitalization		<0.001		<0.001		<0.001
Yes vs. No	5.12 (4.2-6.25)		5.68 (4.33-7.46)		5.79 (4.39-7.63)	
Household size		<0.001*		0.002*		0.003*
4-6 vs. ≤3	0.76 (0.63-0.91)		0.63 (0.49-0.82)		0.64 (0.49-0.83)	
>6 vs. ≤3	1.66 (0.79-3.51)		0.57 (0.22-1.47)		0.54 (0.20-1.43)	
Proportion of members aged over 65		<0.001*		<0.001*		<0.001*
≤0.5 vs.0	2.45 (1.96-3.05)		1.41 (1.05-1.89)		1.44 (1.06-1.94)	
>0.5 vs.0	6.13 (4.64-8.11)		2.48 (1.67-3.7)		2.62 (1.74-3.94)	
Occupation of household head		<0.001		0.008		0.013
Non-farmer vs. Farmer	0.42 (0.27-0.65)		0.76 (0.45-1.27)		0.75 (0.44-1.28)	
Unemployed vs. Farmer	6.51 (4.81-8.81)		1.81 (1.2-2.73)		1.77 (1.16-2.70)	

**Intraclass correlation(ICC)**						0.05

* P-value for trend test was <0.05; Δ Likelihood ratio test; ** ANOVA test

**Table 6:** Determinants of impoverishing health payment

Variables	Univariate analysis		Logistic regression		Mixed effected logistic regression	
	
	Crude OR (95% CI)	P-value^Δ^	Adjusted OR (95% CI)	P-value^Δ^	Adjusted OR (95% CI)	P-value**
**Village-level variables**						
Village deprivation(Quartile)		<0.001*		<0.001*		<0.001*
2nd vs. Lowest deprived	0.9 (0.65-1.25)		1.77 (1.11-2.83)		1.58(0.87-2.86)	
3rd vs. Lowest deprived	0.39 (0.27-0.54)		0.83 (0.52-1.33)		0.81(0.45-1.43)	
Highest vs. Lowest deprived	0.2 (0.14-0.29)		0.39 (0.23-0.64)		0.36(0.20-0.66)	
Availability of health clinic		0.109		0.722		0.7647
Yes vs. No	0.83 (0.66-1.03)		0.94 (0.68-1.3)		1.06(0.70-1.63)	
**Household-level variables**						
Household per capita income (Quartile)		<0.001*		<0.001*		<0.001*
2nd vs. Lowest	0.27 (0.21-0.35)		0.25 (0.18-0.35)		0.25(0.18-0.34)	
3rd vs. Lowest	0.07 (0.05-0.1)		0.07 (0.04-0.1)		0.06(0.04-0.10)	
Highest vs. Lowest	0.02 (0.01-0.04)		0.02 (0.01-0.03)		0.02(0.00-0.03)	
Serious chronic illness proportion		<0.001*		<0.001*		<0.001*
≤0.5 vs.0	5.57 (4.34-7.16)		3.82 (2.81-5.18)		3.88(2.84-5.32)	
>0.5 vs.0	10.33 (7.28-14.64)		6.26 (4-9.79)		6.72(4.23-10.67)	
Member hospitalization		<0.001		<0.001		<0.001
Yes vs. No	4.55 (3.6-5.76)		4.75 (3.44-6.54)		4.94(3.56-6.86)	
Household size		0.123		0.929		0.968
4-6 vs. ≤3	1.02 (0.82-1.28)		0.94 (0.69-1.28)		0.96 (0.70-1.32)	
>6 vs. ≤3	2.39 (1.01-5.67)		0.94 (0.31-2.81)		0.96 (0.31-3.00)	
Proportion of members aged over 65		<0.001*		<0.001*		<0.001*
≤0.5 vs.0	2.37 (1.81-3.1)		1.28 (0.9-1.82)		1.29(0.90-1.86)	
>0.5 vs.0	5.1 (3.59-7.24)		2.79 (1.69-4.6)		3.04(1.81-5.11)	
Occupation of household head		<0.001		0.014		0.013
Non-farmer vs. Farmer	0.3 (0.16-0.57)		0.47 (0.23-0.98)		0.46(0.22-0.97)	
Unemployed vs. Farmer	5.45 (3.78-7.84)		1.61 (0.97-2.68)		1.50(0.88-2.54)	

**Intraclass correlation (ICC)**						0.006

* P-value for trend test was <0.05; Δ Likelihood ratio test; ** ANOVA test

## Discussion

From the current study, village deprivation is an independent predictor for catastrophic and impoverishing health payment after adjusting for household level variables. However, the presence of village health posts, another contextual variable, had no effect on the two outcomes.

Average village income is the most important part of the deprivation score as shown by correlation of these two variables being as high as 0.94. This contextual wealth variable acted independently from individual wealth, which is consistent with other studies [11,25]. For this finding, there are many plausible explanations, including poorer contextual sanitation status, poorer road conditions and limited transportation facilities, and geographic barriers to the collection of the health insurance reimbursement. These contextual disadvantages and inconveniences resulting from deprived village can, finally, lead to an increasing likelihood of financial problems related to health care.

Adult literacy rate in the village has a protective effect on financial difficulties due to health payment and goes independently from individual education level. Reasons for this result can be interpreted as that adult literacy rate is associated with the spreading attitude toward health and health-related behaviors in the same village. Therefore, this contextual variable may be a proxy of the norm of the community in health protection and reaction to health problems.

Remoteness of the village, or geographic barrier, was expressed in this dataset as distance from village to town health center and county hospital. Although this is a part of the deprivation score, the correlations between them is not as strong as the first two contextual variables. Geographic barrier may lead to delay in health seeking [26-29] and consequently followed by more serious health conditions which need more health spending.

The presence of village health clinics had no impact in lowering financial hardship due to health payment. This was different from a finding from a U.S. study[30], which acknowledged that qualified health centers could reduce the odds of high financial burden due to health spending. Owing to poor human resources and facilities, the village health clinics in rural China undertake only the simplest treatment for health care and disease prevention[31], thus resulting in low health payment. Had the quality of care in the village been high, there would have been a lower percentage of catastrophic and impoverishing health payment. Therefore, it is essential to improve the quality of healthcare services offered by the village health posts, especially in the deprived villages.

The New Rural Cooperative Medical Scheme has been conceived as one of the most important mechanisms in reducing the financial burden of health care in rural China[15]. Our study and other studies[13,20] demonstrate clearly that this facility is not reaching the population in need and its implementation, in relation to both the content and level of benefit, and the current mechanism for reaching the target population, needs to be carefully assessed.

As expected, low-income households are more likely than other groups to incur financial catastrophe and impoverishment due to health payment; a finding similar to the experiences of many developing countries in Asia[5,16]. This is mainly due to the limited ability to pay for non-subsistence spending among low-income households, thus more easily incurring catastrophic payment. Additionally, low-income households lay close to the poverty line, hence are easier to fall or descend deeper into poverty, even as a result of low health expenditure.

Similar to reports from other studies[1,7,32], serious chronic illness was also found to be an important determinant of these financial problems caused by health costs. Therefore, it is crucial to pay close attention to whether and how insurance schemes address the specific health needs of the targeted population with serious chronic illness. In addition, the disease control programs in rural China have traditionally focused on infectious diseases; we suggest caution in adopting this method, as the imminent chronic non-communicable disease burden would have a greater impact on catastrophic and impoverishing expenditure.

Having a high proportion of elderly members in a household greatly increases the likelihood of incurring catastrophic and impoverishing health spending; a fact also observed in other studies[6,16]. This finding is associated with the aging population's generally greater need for health care than younger groups[33]. Although the Chinese healthcare system ensures NRCMS coverage and the provision of health services for the elderly, most health expenses need to be paid by the patient[15]. In addition, the Chinese health security system still lacks specific health care services for the elderly, such as long-term care. Solving these problems is particularly important with the elderly increasingly becoming a social issue in rural China[33].

Increasing household size has a protective effect against catastrophic health payment but not against household impoverishment, which is consistent with other studies[34]. This is possibly because larger households can have greater financial support from household members, but the effect of this support is limited in extent and can not prevent impoverishment.

Perhaps the main policy significance of our study is this: In order to maximize the effectiveness of protection against financial hardship due to health spending, health delivery and health financing systems need to target not only people, but also places, since improvements in health and its financial burden in this segment of the population (where both the need for health care and health financial burdens are the most serious) will bring greater overall benefit than would investments that are equally spread across the entire population. Additionally, it is suggested that the health security scheme, which aims to reduce the financial burden caused by health care, should also be linked with the objectives of social development programs, such as the development of the area's economy, improvement to transportation, enhancement of education and reduction of poverty.

This study is based on a large-scale representative household survey with a very high response rate, and is probably the first to explore the contextual effects of the community on financial catastrophe and impoverishment due to health care using a multilevel analysis technique.

However, there are some weaknesses that need to be mentioned. Firstly, there may be a recall bias in the responses to questions regarding health payment and living standards, leading to a certain degree of misclassification. Secondly, the cut-off point for financial catastrophe, proportion of household members with serious chronic disease and family size, were based on arbitrary values, which may be subject to change. However, the threshold of catastrophe set in this study was similar to that previously accepted[35], to make it comparable to other studies. Thirdly, since serious chronic condition was self-reported, which was less objective and precise than direct medical diagnosis, it may result in an overestimation of chronic disease prevalence. Finally, out-of-pocket spending does not take into account the fact that households may choose not to seek care rather than become impoverished, which could also bias the result.

## Competing interests

The authors declare that they have no competing interests.

## Authors' contributions

WS was the main person who designed the study, supervised all fieldwork, analyzed and interpreted the data and drafted the manuscript. VC and AG co-supervised WS during study design, analysis and interpretation of data. VC gave further input in manuscript preparation, which was also contributed to by AG. HZ and DB participated in the design and management of fieldwork. JZ conceptualized the project, managed the coordination and oversaw all implementation activities. All authors read and approved the final manuscript.
