# Effect of household and village characteristics on financial catastrophe and impoverishment due to health care spending in Western and Central Rural China: A multilevel analysis

**DOI:** 10.1186/1478-4505-9-21

**Published:** 2011-06-07

**Authors:** Wuxiang Shi, Virasakdi Chongsuvivatwong, Alan Geater, Hong Zhang, Junhua Zhang, Daniele Brombal, Maria Santonastaso, Giorgio Mario Cortassa

**Affiliations:** 1 Unit of Epidemiology & Health Statistics, Guilin Medical University, Guilin 541004, China; 2 Epidemiology Unit, Faculty of Medicine, Prince of Songkla University, Hat Yai, Songkhla 90110, Thailand; 3 Health Human Resources Development Center, Ministry of Health, Beijing 100097, China; 4 Health Coordination Unit (HCU), Sino-Italian Development Cooperation, Beijing 100097, China

## Correction

After publication of this work [1], we noted that we inadvertently failed to include the complete list of all coauthors. The full list of authors has now been added and the Authors' contributions and Competing interests section modified accordingly.

## Competing interests

The authors declare that they have no competing interests.

## Authors' contributions

WS and DB jointly designed the study and supervised all fieldwork. Additionally, WS analyzed and interpreted the data and drafted the manuscript. VC and AG co-supervised WS during study design, analysis and interpretation of data. VC gave further input in manuscript preparation, which was also contributed to by AG. HZ participated in the design and management of fieldwork. JZ and GC conceptualized the project, managed the coordination and oversaw all implementation activities. MS participated in the design of the study, providing technical and administrative support throughout the entire implementation of the research project. All authors read and approved the final manuscript.
